# U-shaped association between dietary niacin intake and chronic kidney disease among US elderly: a nationwide cross-sectional study

**DOI:** 10.3389/fendo.2024.1438373

**Published:** 2024-10-21

**Authors:** Zhouzhou Xie, Shansen Peng, Gejun Ou, Xiaoqi Zhou, Guihao Zhang, Huiming Jiang, Tianhui Zhang, Nanhui Chen

**Affiliations:** ^1^ Meizhou Clinical Institute of Shantou University Medical College, Meizhou, China; ^2^ Department of Urology, Meizhou People’s Hospital (Meizhou Academy of Medical Sciences), Meizhou, China; ^3^ Departments of Radiology, Meizhou People’s Hospital (Meizhou Academy of Medical Sciences), Meizhou, China

**Keywords:** dietary niacin, chronic kidney disease, NHANES, elderly, u-shaped

## Abstract

**Background:**

In addition to hypertension or diabetes, elderly people are also considered one of the high-risk groups for chronic kidney disease (CKD). Although niacin is recognized for its renal protective properties, the link between dietary niacin intake and CKD remains uncertain. This study investigated this relationship in the elderly.

**Methods:**

We included participants aged 60 and older from the National Health and Nutrition Examination Survey (NHANES) for the years 2003-2018. Dietary niacin intake was assessed through two non-consecutive 24-hour dietary recalls. CKD was diagnosed in individuals with a urine albumin-to-creatinine ratio exceeding 30 mg/g or an estimated glomerular filtration rate below 60 mL/min per 1.73 m^2. The study cohort comprised 4,649 participants, 1,632 of whom had CKD. Propensity score matching (PSM) was utilized to adjust for baseline differences between the groups.

**Results:**

Our analysis, using smooth curve fitting and generalized additive models both before and after PSM, found a U-shaped curve depicting the relationship between dietary niacin intake and CKD risk, confirmed by a log-likelihood ratio test (P < 0.05). Threshold effect analysis (after PSM) indicated a reduced risk of CKD in older adults with a niacin intake below 38.83 mg per day [odds ratio (OR) = 0.99, 95% confidence interval (CI) 0.97-1.00]. In contrast, higher intake levels significantly increased the risk (OR = 1.03, 95% CI 1.00-1.06). Subgroup analysis indicated that these associations were consistent across different stratification variables (P for interaction > 0.05).

**Conclusion:**

Our findings suggested a U-shaped association between dietary niacin intake and CKD risk among older Americans. However, further prospective cohort studies are needed to confirm this finding.

## 1 Introduction

Chronic kidney disease (CKD) constitutes a major public health issue worldwide, afflicting more than 10% of the adult population in the United States and resulting in an annual financial burden exceeding $50 billion (1). Not only does CKD elevate the risk of cardiovascular diseases, but it also leads to renal insufficiency and end-stage renal disease, necessitating interventions such as dialysis or kidney transplantation (2). Individuals aged 60 years and older are particularly susceptible to CKD, a concern that was exacerbated by the aging population dynamics, thereby underlining the critical need for strategies to prevent CKD in the elderly (3, 4).

Among the range of controllable lifestyle factors, dietary habits were particularly crucial. Appropriate nutritional intake can not only mitigate the risk of CKD but also enhances the overall health status of individuals (5). Niacin, or Vitamin B3, is an essential nutrient prevalent in a diverse array of foods such as meats, fish, grains, and dairy products (6). It functions as a precursor for synthesizing nicotinamide adenine dinucleotide (NAD), playing a crucial role in cellular metabolic processes, redox reactions, and energy metabolism (7). Niacin mitigates endothelial dysfunction by elevating intracellular NAD levels, enhancing glutathione synthesis, and diminishing reactive oxygen species production in endothelial cells (8). Current research suggests that supplementation with NAD precursors might represent a promising preventative and therapeutic strategy against renal damage (9, 10). Abnormal levels of myeloperoxidase, lipids and hyperphosphatemia, to varying degrees, indirectly or directly compromise the body’s ability to counteract oxidative stress and inflammation, thereby exacerbating renal dysfunction (11–13). There were indications that niacin could improve dyslipidemia, oxidative stress, inflammatory responses, endothelial function, and serum phosphate levels, thus potentially delaying the progression of CKD (14). Studies suggested that a high dietary intake of niacin might benefit renal function in the elderly to some extent (15). However, some metabolites of niacin, such as prostaglandin D2, homocysteine, and 5-hydroxytryptamine, may also adversely affect renal function. Given niacin’s potential dual effects on kidney health, it is crucial to establish an optimal daily intake range to maximize its protective benefits (16–18).

As far as we know, the association between dietary niacin intake and CKD in the elderly continues to be poorly understood, especially the effects of excessive intake. Considering this, exploring the curvilinear association between the two, and determining the optimal intake threshold, is of paramount importance for shaping public health policies and managing individual health among elderly. In this study, we utilized data spanning from 2003 to 2018 from the National Health and Nutrition Examination Survey (NHANES) to conduct a cross-sectional analysis.

## 2 Materials and methods

### 2.1 Study participants

NHANES, overseen by the National Center for Health Statistics at the Centers for Disease Control and Prevention, utilizes a stratified, multistage sampling approach for its continuous survey efforts. NHANES is a nationwide cross-sectional study. This initiative seeks to thoroughly evaluate the health and nutritional conditions of U.S. adults and children via structured interviews and physical exams. The data required for this analysis were obtained directly from the NHANES official website (https://www.cdc.gov/nchs/nhanes). Written consent was obtained from all NHANES participants for their participation.

**Figure 1:**
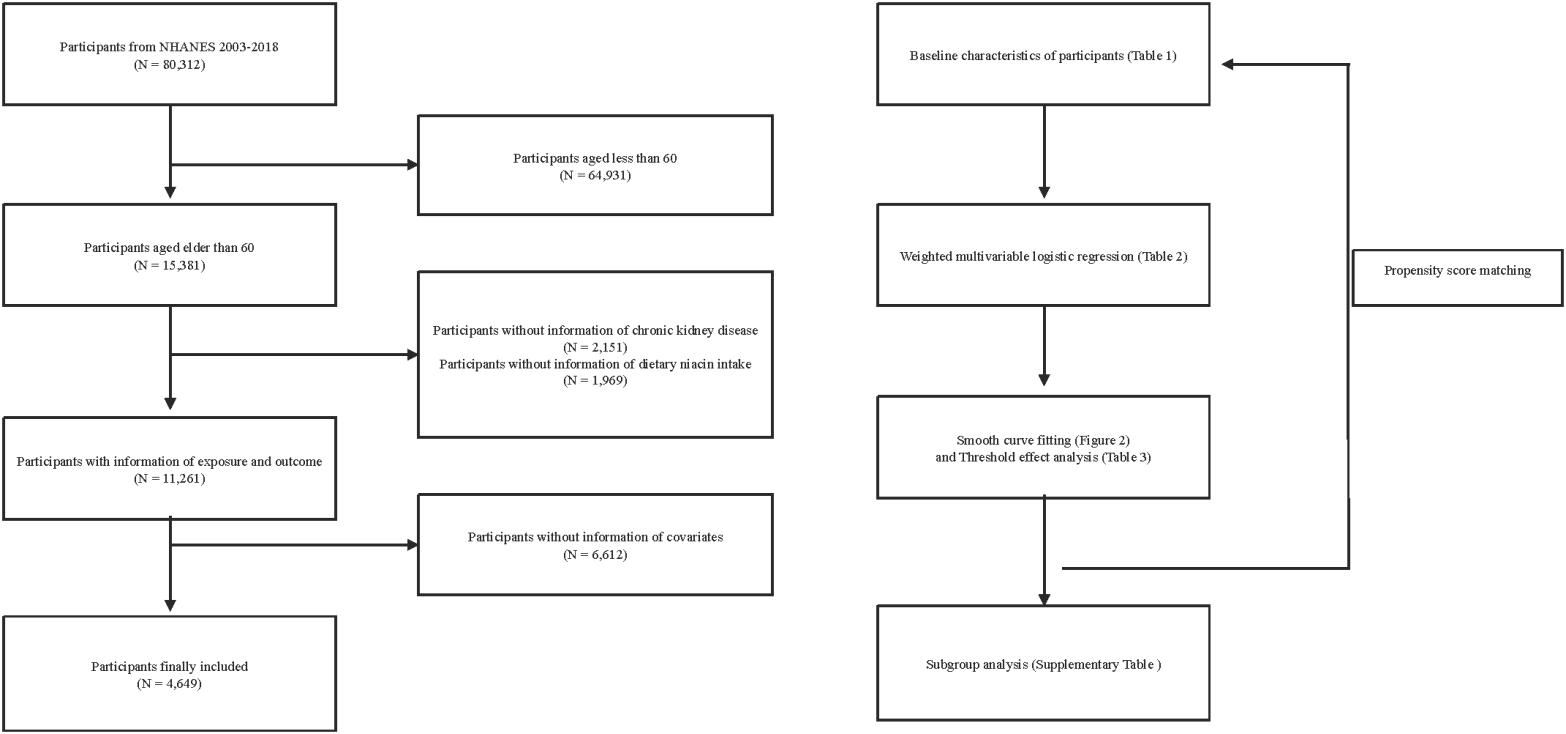
Flow chart for inclusion and exclusion of study participants. NHANES, National Health and Nutrition Examination Survey. .

This study is a cross-sectional analysis utilizing continuous NHANES data collected over eight cycles from 2003 to 2018. Over this 16-year span, an initial pool of 80,312 participants was considered. Firstly, 64,931 individuals under the age of 60 were excluded from the analysis. Subsequently, 2,151 individuals with unclear CKD diagnoses and 1,969 participants with missing dietary niacin intake data were also excluded. Furthermore, 6,612 participants lacking other relevant covariate information were removed from the analysis. Ultimately, this study included 4,649 participants who met all the inclusion criteria (**Figure 1**).

### 2.2 Dietary niacin intake

Dietary niacin intake is evaluated using the dietary interview section of NHANES. All individuals eligible to participate in NHANES are required to complete two rounds of 24-hour dietary recall interviews. These interviews record the dietary intake for the day prior to the interview, from midnight to midnight. To more accurately estimate long-term nutrient intake, it is recommended to compute an average based on at least two non-consecutive days of dietary data (19). This methodological approach enhances the reliability of the nutrient intake estimates by mitigating the day-to-day variability in intake patterns.

### 2.3 Definition of CKD

CKD is diagnosed when at least one of the following criteria is met: 1) a ratio of albumin to creatinine in the urine exceeding 30 mg/g; 2) the estimated glomerular filtration rate (eGFR) estimated to be less than 60 mL/min per 1.73 m^2 (20). The measurement of albumin in the urine utilizes a solid-phase fluorescent immunoassay approach. Creatinine levels in the urine are assessed using the Jaffé method, which involves a kinetic reaction (21). Calculations of eGFR are based on the creatinine formula from the Chronic Kidney Disease Epidemiology Collaboration (CKD-EPI) (22).

### 2.4 Selection of covariates

To mitigate potential confounding factors, this study considered several covariates: age, body mass index (BMI), gender, race, marital status, educational level, poverty index ratio (PIR), smoking status, drinking status, activity, hypertension, diabetes, and various biochemical markers. The biochemical markers analyzed included uric acid, serum phosphate, total cholesterol (TC), triglycerides (TG), low-density lipoprotein cholesterol (LDL-C), and high-density lipoprotein cholesterol (HDL-C).

### 2.5 Statistical analysis

Considering the complexities of the multistage sampling design, we employed the sample weighting data and combination methods recommended by NHANES to ensure national representativeness in our analyses. For baseline characteristics, continuous variables were described using weighted means (95% confidence intervals), while categorical variables were presented as the number of observed (weighted percentage). To assess differences among CKD populations, the study employed weighted linear regression analysis for the appraisal of continuous variables and utilized weighted Chi-square tests to examine categorical variables. To investigate the relationship between dietary niacin intake and CKD, we employed weighted multivariate logistic regression models, establishing three different analytical models: 1) Model 1, which adjusted for no covariates; 2) Model 2, which included preliminary adjustments for age, gender, race, marital status, educational level, and PIR; 3) Model 3, which comprehensively adjusted for all covariates. Initially, dietary niacin intake was examined as a continuous variable. To preliminarily explore potential dose-response relationships, we divided niacin intake into four equally spaced quartiles (Q1, Q2, Q3, Q4) and conducted tests for trend.

To corroborate the robustness of our initial findings, we conducted a series of sensitivity analyses. We utilized smoothing spline fits and generalized additive models (GAM) to probe the non-linear relationship between dietary niacin intake and CKD. GAMs can explore the non-linear relationship between independent and dependent variables through smooth functions, offering greater expressive power than linear models (23). Based on previous literature, we set the smoothness parameter to 3 to ensure the curve’s smoothness (24). Subsequently, a piecewise linear regression approach was adopted to delineate two distinct segments and compute threshold effects. We contrasted this with a traditional linear model, applying a Log-likelihood ratio test to verify the presence of any thresholds. The threshold size was identified using a two steps recursive method (25). Additionally, subgroup analyses were conducted to explore whether the impact of dietary niacin intake on CKD varied across different strata, assessing interaction effects through likelihood ratio tests. Lastly, given the baseline differences between CKD and non-CKD participants (**Table 1**), propensity score matching (PSM) was used to align baseline characteristics between the two groups, thereby controlling for confounding and preventing bias. All baseline characteristics in **Table 1**, except for dietary niacin intake, were included in the PSM model, employing a 1:1 nearest neighbor matching technique with a caliper width allowing for a 0.05 difference in propensity scores. A smaller caliper width helps to improve the balance between matching pairs, thus better controlling for the effect of confounding variables (26).

**Table 1:** Baseline characteristics of participants between 2003 and 2018 before and after PSM.

Characteristic	Before PSM			After PSM		
	Non-CKD	CKD	P	Non-CKD	CKD	P
	(N = 3017)	(N = 1632)		(N = 1369)	(N = 1369)	
**Age, years**	67.82 (67.54, 68.11)	72.74 (72.28, 73.20)	<0.0001	70.47 (69.99, 70.95)	71.89 (71.39, 72.38)	<0.0001
**BMI, kg/m^2^**	29.00 (28.67, 29.33)	29.65 (29.25, 30.04)	0.0125	29.13 (28.71, 29.55)	29.52 (29.08, 29.96)	0.1734
**Gender, n (%)**			0.0158			0.0794
Male	1506 (46.46)	786 (41.59 )		689 (47.03)	673 (42.81)	
Female	1511 (53.54)	846 (58.41)		680 (52.97)	696 (57.19)	
**Race, n (%)**			0.4038			0.7087
Mexican American	694 (6.84)	276 (6.19)		278 (6.80)	241 (6.35)	
Non-Hispanic White	1582 (81.40)	990 (81.15)		750 (79.37)	812 (80.66)	
Non-Hispanic Black	558 (7.58)	285 (8.68)		254 (8.88)	243 (8.67)	
Other	183 (4.17)	81 (8.68)		87 (4.95)	73 (4.31)	
**Marital status, n (%)**			<0.0001			0.015
Married/with partner	1934 (69.83)	912 (58.23)		822 (65.99)	796 (60.79)	
Single	1083 (30.17)	720 (41.77)		547 (34.01)	573 (39.21)	
**Education level, n (%)**			<0.0001			0.3031
Less than high school	812 (15.98)	498 (21.03)		410 (20.75)	409 (20.30)	
High school or equivalent	720 (24.62)	446 (29.94)		315 (24.61)	349 (28.07)	
College or above	1485 (59.40)	688 (49.04 )		644 (54.64)	611 (51.63)	
**PIR, n (%)**			<0.0001			0.682
< = 1.30	750 (14.05)	465 (19.46)		375 (17.18)	385 (18.84)	
> 1.30, < = 3.50	1259 (39.39)	762 (47.17)		620 (46.78)	625 (45.58)	
> 3.50	1008 (46.56)	405 (33.37)		374 (36.04)	359 (35.58)	
**Smoking status, n (%)**			0.7			0.5159
No	1420 (47.34)	798 (48.19)		648 (45.87)	668 (47.57)	
Yes	1597 (52.66)	834 (51.81)		721 (54.13)	701 (52.43)	
**Drinking status, n (%)**			<0.0001			0.1035
No	876 (25.20 )	580 (34.13)		436 (28.75)	467 (32.52)	
Yes	2141 (74.80)	1052 (65.87)		933 (71.25)	902 (67.48)	
**Activity, n (%)**			<0.0001			0.1156
Inactive or moderate	2654 (86.28)	1535 (93.16)		1239 (89.88)	1277 (92.22)	
Vigorous	363 (13.72)	97 (6.84)		130 (10.12)	92 (7.78)	
**Hypertension, n (%)**			<0.0001			0.0075
No	1365 (47.80)	445 (27.09)		482 (36.92)	424 (30.60)	
Yes	1652 (52.20)	1187 (72.91)		887 (63.08)	945 (69.40)	
**Diabetes, n (%)**			<0.0001			0.0003
No	2391 (82.82)	1047 (68.28)		990 (76.20)	908 (70.99)	
Yes	525 (13.93)	535 (28.29)		306 (18.70)	426 (26.13)	
Borderline	101 (3.24)	50 (3.43)		73 (5.10)	35 (2.88)	
**Uric acid, mg/dL**	5.46 (5.40, 5.53)	6.27 (6.18, 6.36)	<0.0001	5.76 (5.67, 5.86)	6.03 (5.94, 6.12)	0.0003
**Phosphorus, mg/dL**	3.63 (3.59, 3.66)	3.72 (3.69, 3.76)	0.0004	3.65 (3.61, 3.68)	3.70 (3.66, 3.74)	0.0376
**HDL-C, mg/dL**	57.54 (56.54, 58.53)	55.73 (54.34, 57.12)	0.0257	56.27 (55.21, 57.33)	56.54 (54.96, 58.81)	0.7634
**LDL-C, mg/dL**	115.12 (113.36, 116.88)	106.13 (103.72, 108.55)	<0.0001	111.55 (109.39, 113.71)	107.49 (104.73, 110.25)	0.0177
**TG, mg/dL**	122.21 (118.25, 126.16)	130.90 (126.39, 135.41)	0.0035	127.93 (123.06 ,132.80)	128.54 (123.55 ,133.53)	0.8644
**TC, mg/dL**	197.09 (194.97, 199.21)	188.04 (185.26, 190.82)	<0.0001	193.40 (190.84 ,195.95)	189.73 (186.54, 192.92)	0.0588
**Dietary niacin intake, mg**	23.76 (23.02, 24.50)	20.78 (20.12, 21.43)	<0.0001	22.34 (21.69, 22.99)	21.03 (20.26, 21.79)	0.0025

CKD, chronic kidney disease; PSM, propensity score matching; BMI, body mass index; PIR, poverty income ratio; HDL-C, high-density lipoprotein cholesterol; LDL-C, low-density lipoprotein cholesterol; TG, triglyceride; TC, total cholesterol; N/n, number of observed; %, survey-weighted percentage.

For continuous variables: numerical value with survey-weighted mean (95% CI), P was by sur-vey-weighted linear regression.

For categorical variables: number of observed with survey-weighted percentage, P was by sur-vey-weighted Chi-square test.

R software (version 4.3.2) and EmpowerStats (X&Y Solutions, Inc., Boston, MA) were the tools utilized for the statistical computations and graphical representations in this study. We defined statistical significance at a cutoff of p<0.05 using a two-sided test.

## 3 Results

### 3.1 Baseline characteristics of participants

Our research encompassed 4,649 individuals aged 60 and above, all subject to detailed inclusion and exclusion criteria outlined (**Figure 1**). Within this group, 1,632 were identified as having CKD. Before PSM, significant statistical differences were observed across most baseline characteristics between the groups categorized by CKD status (**Table 1**). After applying a 1:1 PSM, the number of participants reduced to 2,738, evenly distributed between the CKD and non-CKD groups. After PSM, significant reductions were observed in the statistical differences between the two groups regarding BMI, gender, educational level, PIR, drinking status, activity, HDL-C, TG, and TC.

### 3.2 Associations between dietary niacin intake and CKD in older adults

Findings from the logistic regression revealed a notable inverse correlation between dietary niacin intake and the occurrence of CKD among older adults (**Table 2**). The link was statistically significant in the unadjusted Model 1 [odds ratio (OR) = 0.97, 95% confidence interval (CI) 0.96-0.98], showed persistence in the minimally adjusted Model 2 (OR = 0.98, 95% CI 0.97-0.99), and remained stable in the fully adjusted Model 3 (OR = 0.99, 95% CI 0.97-1.00). The robustness of these findings was corroborated by the Model 4 (after PSM: OR = 0.99, 95% CI 0.98-1.00).

**Table 2:** Before and after PSM, weighted multivariable logistic regression for the association between dietary niacin intake and CKD.

Before PSM	OR (95%CI)			After PSM	OR (95%CI)
	Model 1	Model 2	Model 3		Model 4
Niacin continuous, mg	0.97 (0.96, 0.98)	0.98 (0.97, 0.99)	0.99 (0.97, 1.00)	Niacin continuous, mg	0.99 (0.98, 1.00)
Niacin quartile, mg				Niacin quartile, mg	
Q1 ( < 14.90)	1.00 (reference)	1.00 (reference)	1.00 (reference)	Q1 ( < 14.58)	1.00 (reference)
Q2 (14.90 - 20.09)	0.83 (0.66, 1.03)	0.92 (0.72, 1.18)	0.86 (0.66, 1.13)	Q2 (14.59 - 19.68)	0.80 (0.61, 1.06)
Q3 (21.10 - 26.38)	0.57 (0.45, 0.71)	0.66 (0.51, 0.86)	0.64 (0.48, 0.85)	Q3 (19.69 - 25.95)	0.64 (0.47, 0.89)
Q4 ( > 26.38)	0.48 (0.38, 0.62)	0.67 (0.51, 0.89)	0.70 (0.51, 0.95)	Q4 ( > 25.96)	0.68 (0.51, 0.90)
P for trend	<0.0001	0.0012	0.0075	P for trend	0.0043

OR: odds ratio; 95%CI: 95% conﬁdence interval; Q: quartile; PSM: propensity score matching.

Model 1: No covariates were adjusted.

Model 2: Age, gender, race, education level, marital status, and PIR were adjusted.

Model 3: Age, gender, race, educational level, marital status, PIR, smoking status, drinking status, activity, hypertension, diabetes, BMI, uric acid, phosphorus, HDL-C, LDL-C, TG and TC were adjusted.

Model 4: After PSM, the covariates were adjusted in accordance with model 3.

In Model 3, a negative association was found between dietary niacin intake and CKD when the lowest quartile of intake was used as a reference. OR for the second, third, and highest quartiles were 0.86 (95% CI 0.66-1.13), 0.64 (95% CI 0.48-0.85), and 0.70 (95% CI 0.51-0.95) respectively, with a trend significance (P for trend = 0.0075). It is of particular interest that the most pronounced protective effect of niacin was evident in the third quartile, implying a potential threshold effect. This pattern aligns with results from the Model 4, suggesting a non-linear link between niacin and CKD risk among the elderly.

### 3.3 The non-linear relationship between dietary niacin intake and CKD prevalence in older adults

**Figure 2:**
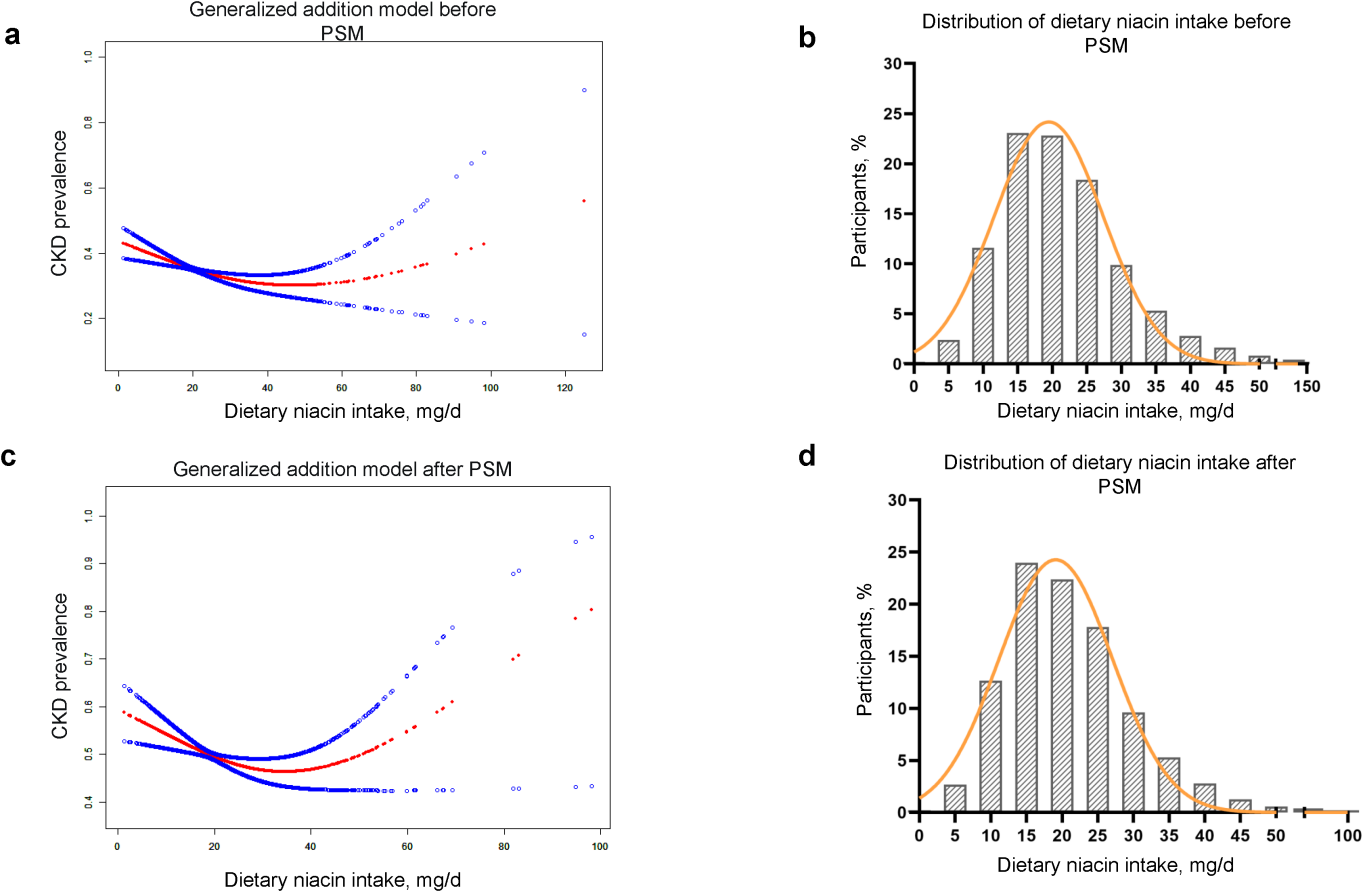
U-shape association of the dietary niacin intake and CKD prevalence before and after PSM. **(A, B)** Smooth curve fitting and frequency distribution before PSM. **(C, D)** Smooth curve fitting and frequency distribution after PSM. PSM, propensity score matching; CKD, chronic kidney disease. Age, gender, race, educational level, marital status, PIR, smoking status, drinking status, activity, hypertension, diabetes, BMI, uric acid, phosphorus, HDL-C, LDL-C, TG and TC were adjusted.

After multivariate adjustments in Model 3, smoothing spline fitting and GAM elucidated a U-shaped association between these variables (**Figures 2A, C**). Using a two-piecewise linear regression model, we demonstrated a significant non-linear relationship between dietary niacin intake and CKD risk both before and after PSM (P for log likelihood ratio test < 0.05) in **Table 3**.

**Table 3:** Threshold effect analysis using two-piecewise linear regression based on dietary niacin intake and CKD before and after PSM.

Dietary niacin intake, mg	OR (95%CI)	P
**Before PSM**		
Fitting model by two-piecewise linear regression		
Inflection point		
< 40.35	0.99 (0.98, 1.00)	0.0054
> = 40.35	1.02 (0.99, 1.04)	0.1563
P for log likelihood ratio test		0.04
**After PSM**		
Fitting model by two-piecewise linear regression		
Inflection point		
< 38.83	0.99 (0.97, 1.00)	0.0055
> = 38.83	1.03 (1.00, 1.06)	0.0426
P for log likelihood ratio test		0.007

PSM, propensity score matching; OR, odds ratio; 95%CI, 95% conﬁdence interval.

Age, gender, race, educational level, marital status, PIR, smoking status, drinking status, activity, hypertension, diabetes, BMI, uric acid, phosphorus, HDL-C, LDL-C, TG and TC were adjusted.

The turning points of dietary niacin intake were calculated using the two steps recursive method, identified at 40.35 mg/d before PSM and 38.83 mg/d after PSM. For most participants, daily dietary niacin intake was below these turning points (**Figures 2B, D**). Before reaching these turning points, an increase in dietary niacin intake was significantly associated with a reduced risk of CKD (before PSM: OR = 0.99, 95%CI 0.98-1.00; after PSM: OR = 0.99, 95%CI 0.97-1.00). However, beyond these turning points, the relationship inverted, and higher dietary niacin intake appeared to correlate with an increased risk of CKD, particularly after PSM where the association became more pronounced (OR = 1.03, 95%CI 1.00-1.06).

### 3.4 Subgroup analysis

Further investigation through subgroup analysis was conducted to explore if this curvilinear association differed across various participant characteristics. The findings, as detailed in **Supplementary Table 1**, were very stable, showing no significant interactions in any stratification variables (P for interaction > 0.05). Consistency in results was maintained after PSM, as indicated in **Supplementary Table 2**.

## 4 Discussion

In this nationally representative cross-sectional study, a U-shaped relationship was identified between dietary niacin intake and CKD prevalence among individuals aged 60 and older. We determined the inflection points for dietary niacin intake to be 40.35 mg/d before PSM and 38.83 mg/d after PSM. These thresholds were near the upper intake level of 35 mg/d recommended by the Institute of Medicine for adults (27), suggesting a close alignment with our findings. However, it was notable that the majority of elderly individuals did not reach these niacin intake thresholds. The findings of this study have potential implications for clinical practice, aiding doctors in recommending appropriate niacin intake for elderly. It is crucial to avoid excessive or insufficient niacin intake to maintain a safe range. For public health, these findings underscore the importance of building public awareness about appropriate nutrient intake, developing policies for dietary supplement regulation to prevent excessive intake, updating dietary guidelines for elderly, and emphasizing both upper and lower limits of niacin intake to prevent CKD and other related health issues. In conclusion, this study not only informs individual clinical practice but also provides an empirical basis for public health policy-making. This finding can help reduce the prevalence of CKD in elderly and improve overall health.

Niacin has been reported to contribute to the slowing of eGFR decline, demonstrating significant potential as a renal protective agent (14). However, research linking dietary niacin intake with CKD risk remained limited. A study involving children demonstrated a noteworthy positive correlation between dietary niacin intake and eGFR (28). However, the study did not quantify the niacin intake, and the sample size was limited to 19 participants. The results suggest a potential association between niacin and kidney function in children, but not in the elderly. Moreover, a recent cross-sectional study conducted in Japan discovered a significant association between higher dietary niacin intake and reduced CKD risk among individuals aged 40 and older who were homozygous for the rs883484 allele (OR = 0.74, 95% CI 0.57–0.96) (29).. However, the findings are specific to individuals homozygous for the RS883484 allele, who constitute only 15% of the population. It is also worth noting that the study participants had a mean age of 62.1 ± 10.8 years, which somewhat represents the older population. To a certain extent, this supported some of our findings and underscored the potential preventive benefits of dietary niacin against CKD. Additionally, this study introduced the concept of dietary niacin indications for specific genetic types, which could be an interesting direction for future research. Earlier prospective cohort studies also aligned with our findings, demonstrating that higher dietary niacin intake (14.7 mg/d) was associated with a reduced risk of renal function decline in individuals aged 65 and over compared to the lowest quartile (7.9 mg/d) [Relative Risk (RR) = 0.728, 95% CI 0.548–0.966] over an average follow-up of 3.2 years, highlighting the benefits of niacin for elderly renal function (15). However, compared to our study, the baseline levels of dietary niacin intake were relatively low in this research. For instance, the lowest quartile had an intake of 7.9 mg/d and the highest 14.7 mg/d, which might explain why the previous study did not observe significant threshold effects associated with excessive niacin intake.

Our study offered a new insight into the prevention of CKD among individuals aged 60 and older. We found that for participants with dietary niacin intake below the threshold of 38.83 mg/d (after PSM), an increase in niacin intake was significantly associated with a decreased risk of CKD (OR = 0.99, 95% CI 0.97-1.00). The pathogenesis of CKD was mediated by various risk factors such as oxidative stress, inflammatory responses, and endothelial dysfunction (30). Research in animal models of CKD demonstrated that niacin could mitigate conditions like hypertension, proteinuria, and glomerulosclerosis in mice, reducing kidney oxidative stress, inflammation, and tissue damage (31). Additionally, niacin assisted in mitigating endothelial dysfunction by elevating cellular NAD levels, replenishing glutathione, and decreasing reactive oxygen species production in endothelial cells (8). Recent studies had established a close link between disruptions in NAD synthesis and kidney damage (32), suggesting that modulating NAD metabolism could be a novel therapeutic approach to combat renal cell damage (9). Furthermore, myeloperoxidase (MPO) served as a critical mediator linking oxidative stress, inflammation, and endothelial dysfunction. Elevated MPO activity was linked to the advancement of glomerular lesions (11). Niacin effectively inhibited neutrophil MPO release and treated MPO-mediated inflammatory lesions (33). An increasing number of prospective cohort studies had demonstrated an association between diverse abnormalities in the lipid profile and the progression of CKD (14). This association was particularly significant given the kidney’s highly vascular nature; lipid abnormalities generally exerted harmful effects on renal function. Abnormal lipid levels could impair the body’s antioxidative and anti-inflammatory capacities, subsequently damaging renal vascular endothelial function (12). In summary, dyslipidemia was identified as a predictive factor for the progression of CKD. Niacin had been proven to reduce lipid and serum phosphate levels in the short term, improving the eGFR of CKD patients (34). However, further research is required to evaluate the long-term effects of niacin. Serum phosphate might play an independent pathogenic role in the progression of CKD (13). Hyperphosphatemia was linked with several key regulators of renal vascular calcification, such as elevated levels of fibroblast growth factor 23 (FGF23) and decreased expression of Klotho (35). Niacin not only reduced phosphate absorption in the gut but also promoted its excretion in the urine, helping to control serum phosphate levels (36). A randomized controlled trial confirmed that niacin significantly reduced serum phosphate levels by approximately 0.40 mg/dL, regardless of whether eGFR was <60 or ≥60 ml/min compared to placebo (37). Additional studies also indicated that niacin could significantly improve serum phosphate levels in CKD patients, providing strong evidence for niacin’s role in ameliorating the progression of CKD (34, 38, 39).

In participants with dietary niacin intake exceeding 38.83 mg/d (after PSM), an increase in niacin intake was associated with a significant rise in CKD risk (OR = 1.03, 95%CI 1.00-1.06). This might be attributed to increased levels of metabolites such as prostaglandin D2 (PGD2) (16), homocysteine (HCY) (17), and serotonin (5-HT) (18) following excessive niacin intake. PGD2 was reported to promote renal fibrosis in CKD via CRTH2-mediated activation of Th2 lymphocytes (40). A prospective cohort study identified elevated serum homocysteine (HCY) levels as an independent risk factor for CKD in the general population (41). Hyperhomocysteinemia mediated several key pathogenic mechanisms in CKD, such as oxidative stress, endoplasmic reticulum stress, inflammation, and hypomethylation (42). Afolabi et al. found that 5-HT impaired renal function by activating the transient receptor potential vanilloid 4 channels in smooth muscle cells of the renal microvasculature (43). Using antagonists to inhibit 5-HT expression helped reduce renal fibrosis and inflammation (44). Additionally, recent research indicated that the end metabolite produced from excessive niacin intake, N1-methyl-4-pyridone-3-carboxamide, had biological activity and could cause vascular inflammation and leukocyte adhesion (45). This metabolite was previously considered to be an obsolete uremic toxin (46). Therefore, when consuming dietary niacin, it was crucial to consider the dosage to avoid potential renal side effects. Our study identified a threshold of 38.83 mg/d (after PSM), closely aligning with the tolerable upper intake limit set at 35 mg/d (27). However, considering the adverse effects of exceeding the tolerable intake limit, this threshold might need to be optimized.

Our study had several strengths. Firstly, it was the first to comprehensively assess the association between dietary niacin intake and CKD among the elderly. Secondly, our research considered the complex sampling design and weights of NHANES, ensuring that the data was representative of the national population. Additionally, we adjusted for multiple covariates and used PSM to minimize bias from confounding factors. Moreover, to check the stability of the results, we conducted subgroup analyses. Finally, we fitted smoothing curves and identified a nonlinear relationship between dietary niacin intake and CKD, including determining the threshold effects through analysis.

Nonetheless, our study also encountered some limitations. Firstly, its cross-sectional design precluded definitive establishment of causality. Secondly, despite obtaining dietary data through two 24-hour dietary recalls, the potential for recall bias could not be entirely eliminated. Niacin is predominantly found in animal-derived foods; however, the absence of specific data on vegetarian dietary patterns in the NHANES may introduce a degree of bias that could potentially impact the results. Moreover, there might have been confounding factors that were not fully accounted for. These limitations may impact the accuracy of the study’s findings. However, as a preliminary study, this work will provide a theoretical foundation for future prospective research. Lastly, our study was limited to the population aged 60 and over in the United States and extrapolating the results to other populations necessitates further investigation. In the future, prospective cohort studies of dietary niacin intake in different populations could be conducted to explore the effects of niacin on CKD.

## 5 Conclusions

Our study demonstrates a U-shaped relationship between dietary niacin intake and CKD among the U.S. population aged 60 and older, with a curve inflection point at 38.83 mg/d. This finding offers new perspectives and scientific evidence for early prevention and intervention strategies for CKD in elderly Americans. However, due to the inherent limitations of the cross-sectional study design, it is important to interpret the findings cautiously, as they cannot establish causality or long-term effects. Therefore, further validation through large-scale prospective cohort studies is needed.

## Data Availability

The original contributions presented in the study are included in the article/**Supplementary Material**. Further inquiries can be directed to the corresponding author/s.
