# Supplementary material for: U-shaped association between dietary niacin intake and chronic kidney disease among US elderly: a nationwide cross-sectional study
Source: Front Endocrinol (Lausanne). 2024 Oct 21;15:1438373. doi: 10.3389/fendo.2024.1438373 (PMC11532146; doi:10.3389/fendo.2024.1438373)
Supplement: Supplementary file 1 [file Table1.docx]

Supplementary Table 1: Associations of dietary niacin intake with CKD in various subgroups before PSM.

| **Subgroup** | **Dietary niacin intake quartile(mg/d), OR (95%CI)** | | | | **P for interaction** |
| --- | --- | --- | --- | --- | --- |
|  | **Q1 ( < 14.90)** | **Q2 (14.90 - 20.09)** | **Q3 (21.10 - 26.38)** | **Q4 ( > 26.38)** |  |
| **BMI, kg/m2** |  |  |  |  | 0.8241 |
| < 25 | 1.00 (reference) | 0.99 (0.59, 1.65) | 0.68 (0.39, 1.19) | 0.87 (0.50, 1.52) |  |
| >= 25 | 1.00 (reference) | 0.83 (0.60, 1.14) | 0.62 (0.45, 0.86) | 0.65 (0.45, 0.93) |  |
| **Gender** |  |  |  |  | 0.9626 |
| Male | 1.00 (reference) | 0.91 (0.60, 1.37) | 0.62 (0.42, 0.91) | 0.71 (0.46, 1.08) |  |
| Female | 1.00 (reference) | 0.84 (0.61, 1.16) | 0.66 (0.46, 0.94) | 0.69 (0.45, 1.04) |  |
| **Race** |  |  |  |  | 0.0940 |
| Mexican American | 1.00 (reference) | 0.98 (0.57, 1.68) | 1.02 (0.64, 1.62) | 1.15 (0.72, 1.85) |  |
| Non-Hispanic white | 1.00 (reference) | 0.93 (0.67, 1.28) | 0.63 (0.45, 0.89) | 0.66 (0.46, 0.97) |  |
| Non-Hispanic Black | 1.00 (reference) | 0.56 (0.34, 0.91) | 0.61 (0.36, 1.02) | 0.83 (0.48, 1.43) |  |
| Other | 1.00 (reference) | 0.38 (0.13, 1.17) | 0.40 (0.14, 1.14) | 0.67 (0.22, 2.07) |  |
| **Smoking status** |  |  |  |  | 0.9802 |
| No | 1.00 (reference) | 0.91 (0.62, 1.34) | 0.66 (0.45, 0.97) | 0.73 (0.47, 1.12) |  |
| Yes | 1.00 (reference) | 0.82 (0.56, 1.19) | 0.61 (0.42, 0.89) | 0.67 (0.45, 0.99) |  |
| **Drinking status** |  |  |  |  | 0.9002 |
| No | 1.00 (reference) | 0.79 (0.56, 1.11) | 0.62 (0.40, 0.95) | 0.61 (0.42, 0.90) |  |
| Yes | 1.00 (reference) | 0.92 (0.62, 1.37) | 0.66 (0.45, 0.97) | 0.74 (0.49, 1.12) |  |
| **Activity** |  |  |  |  | 0.1744 |
| Inactive or moderate | 1.00 (reference) | 0.83 (0.62, 1.09) | 0.66 (0.49, 0.89) | 0.67 (0.49, 0.91) |  |
| Vigorous | 1.00 (reference) | 1.44 (0.51, 4.02) | 0.38 (0.13, 1.11) | 1.01 (0.44, 2.30) |  |
| **Hypertension** |  |  |  |  | 0.1753 |
| No | 1.00 (reference) | 0.89 (0.56, 1.40) | 0.46 (0.29, 0.75) | 0.62 (0.39, 0.99) |  |
| Yes | 1.00 (reference) | 0.85 (0.63, 1.15) | 0.74 (0.55, 1.01) | 0.74 (0.51, 1.06) |  |
| **Diabetes** |  |  |  |  | 0.1141 |
| No | 1.00 (reference) | 0.90 (0.66, 1.24) | 0.62 (0.45, 0.87) | 0.68 (0.48, 0.95) |  |
| Yes | 1.00 (reference) | 0.65 (0.40, 1.05) | 0.66 (0.36, 1.20) | 0.62 (0.36, 1.06) |  |
| Borderline | 1.00 (reference) | 1.99 (0.46, 8.61) | 0.65 (0.19, 2.18) | 2.60 (0.84, 7.99) |  |

BMI: body mass index. Each stratification adjusted for all the factors (age, gender, race, educational level, marital status, PIR, smoking status, drinking status, activity, hypertension, diabetes, BMI, uric acid, phosphorus, HDL-C, LDL-C, TG and TC) except the stratification factor itself in model.
