# Supplementary material for: U-shaped association between dietary niacin intake and chronic kidney disease among US elderly: a nationwide cross-sectional study
Source: Front Endocrinol (Lausanne). 2024 Oct 21;15:1438373. doi: 10.3389/fendo.2024.1438373 (PMC11532146; doi:10.3389/fendo.2024.1438373)
Supplement: Supplementary file 2 [file Table2.docx]

Supplementary Table 2: Associations of niacin intake with CKD in various subgroups after PSM.

| **Subgroup** | **Niacin intake quartile(mg), OR (95%CI)** | | | | **P for interaction** |
| --- | --- | --- | --- | --- | --- |
|  | **Q1 (< 14.58)** | **Q2 (14.59 - 19.68)** | **Q3 (19.69 - 25.95)** | **Q4 (> 25.96)** |  |
| **BMI, kg/m2** |  |  |  |  | 0.6931 |
| < 25 | 1.00 (reference) | 0.70 (0.40, 1.23) | 0.73 (0.40, 1.32) | 0.76 (0.44, 1.30) |  |
| >= 25 | 1.00 (reference) | 0.65 (0.44, 0.95) | 0.63 (0.43, 0.93) | 0.56 (0.38, 0.83) |  |
| **Gender** |  |  |  |  | 0.8049 |
| Male | 1.00 (reference) | 0.77 (0.48, 1.23) | 0.71 (0.45, 1.11) | 0.72 (0.49, 1.07) |  |
| Female | 1.00 (reference) | 0.83 (0.59, 1.17) | 0.61 (0.41, 0.89) | 0.64 (0.41, 1.01) |  |
| **Race** |  |  |  |  | 0.2272 |
| Mexican American | 1.00 (reference) | 1.33 (0.71, 2.48) | 0.99 (0.58, 1.67) | 1.18 (0.70, 1.99) |  |
| Non-Hispanic white | 1.00 (reference) | 0.83 (0.58, 1.17) | 0.63 (0.42, 0.93) | 0.64 (0.45, 0.90) |  |
| Non-Hispanic Black | 1.00 (reference) | 0.59 (0.36, 0.98) | 0.65 (0.37, 1.14) | 0.84 (0.43, 1.63) |  |
| Other | 1.00 (reference) | 0.36 (0.12, 1.06) | 0.43 (0.14, 1.36) | 0.53 (0.17, 1.62) |  |
| **Smoking status** |  |  |  |  | 0.2649 |
| No | 1.00 (reference) | 0.65 (0.43, 0.97) | 0.66 (0.42, 1.02) | 0.67 (0.42, 1.05) |  |
| Yes | 1.00 (reference) | 1.04 (0.70, 1.54) | 0.64 (0.43, 0.94) | 0.70 (0.49, 1.00) |  |
| **Drinking status** |  |  |  |  | 0.7644 |
| No | 1.00 (reference) | 0.65 (0.44, 0.98) | 0.59 (0.35, 0.98) | 0.63 (0.40, 0.99) |  |
| Yes | 1.00 (reference) | 0.92 (0.60, 1.39) | 0.69 (0.46, 1.05) | 0.73 (0.50, 1.05) |  |
| **Activity** |  |  |  |  | 0.1105 |
| Inactive or moderate | 1.00 (reference) | 0.78 (0.58, 1.06) | 0.70 (0.50, 0.98) | 0.67 (0.50, 0.90) |  |
| Vigorous | 1.00 (reference) | 0.98 (0.37, 2.56) | 0.19 (0.05, 0.70) | 0.66 (0.27, 1.60) |  |
| **Hypertension** |  |  |  |  | 0.2611 |
| No | 1.00 (reference) | 0.83 (0.52, 1.34) | 0.49 (0.30, 0.82) | 0.57 (0.34, 0.93) |  |
| Yes | 1.00 (reference) | 0.79 (0.56, 1.12) | 0.73 (0.52, 1.02) | 0.74 (0.54, 1.03) |  |
| **Diabetes** |  |  |  |  | 0.5438 |
| No | 1.00 (reference) | 0.85 (0.61, 1.19) | 0.66 (0.45, 0.96) | 0.64 (0.46, 0.90) |  |
| Yes | 1.00 (reference) | 0.60 (0.36, 1.00) | 0.59 (0.29, 1.20) | 0.67 (0.39, 1.15) |  |
| Borderline | 1.00 (reference) | 1.45 (0.24, 8.61) | 0.58 (0.09, 3.74) | 2.28 (0.48, 10.94) |  |

BMI: body mass index. Each stratification adjusted for all the factors (age, gender, race, educational level, marital status, PIR, smoking status, drinking status, activity, hypertension, diabetes, BMI, uric acid, phosphorus, HDL-C, LDL-C, TG and TC) except the stratification factor itself in model.
